# Risk-adjusted policies to minimise perioperative staffing shortages during a pandemic: An agent-based simulation study

**DOI:** 10.1017/S0950268823000511

**Published:** 2023-04-03

**Authors:** Vishnunarayan G. Prabhu, William R. Hand, Tugce Isik, Yongjia Song, Kevin M. Taaffe

**Affiliations:** 1 Industrial and Systems Engineering and Engineering Management, The University of North Carolina at Charlotte, Charlotte, NC, USA; 2 Department of Anesthesiology, Prisma Health – Upstate, Greenville, SC, USA; 3 Department of Industrial Engineering, Clemson University, Clemson, SC, USA

**Keywords:** COVID-19, health policy, infectious disease control, modelling, public health

## Abstract

Healthcare workers’ (HCWs) safety and availability to care for patients are critical during a pandemic such as the one caused by severe acute respiratory syndrome coronavirus 2. Among providers of different specialities, it is critical to protect those working in hospital settings with a high risk of infection. Using an agent-based simulation model, various staffing policies were developed and simulated for 90 days using data from the largest health systems in South Carolina. The model considers staffing policies that include geographic segregation, interpersonal contact limits, and a combination of factors, including the patient census, transmission rates, vaccination status of providers, hospital capacity, incubation time, quarantine period, and interactions between patients and providers. Comparing the existing practices to various risk-adjusted staffing policies, model predictions show that restricted teaming and rotating schedules significantly (*p*-value <0.01) reduced weekly HCW unavailability and the number of infected HCWs by 22% and 38%, respectively, when the vaccination rates among HCWs were lower (<75%). However, as the vaccination rate increases, the benefits of risk-adjusted policies diminish; and when 90% of HCWs were vaccinated, there were no significant (*p*-value = 0.09) benefits. Although these simulated outcomes are specific to one health system, our findings can be generalised to other health systems with multiple locations.

## Introduction

Since its outbreak, severe acute respiratory syndrome coronavirus 2 (SARS-CoV-2) has infected more than 100 million Americans and resulted in 1.1 million deaths in the USA [1]. According to the US Department of Health and Human Services (HHS) report from January 2022, an estimated 141,385 hospital beds were occupied by patients with coronavirus disease 2019 (COVID-19), approaching the record peak from January 2021 [2]. Additionally, the HHS report observed that 25% of US hospitals were facing a critical staffing shortage, which was the highest since the beginning of the COVID-19 pandemic [3, 4].

The perioperative team is considered a high-risk group for COVID-19 exposure as they come in close contact with a patient’s airway and aerosols generated during certain medical procedures where the SARS-CoV-2 virus remains infectious for hours [5–8]. Staffing shortages among perioperative teams lead to delayed patient care and poorer healthcare outcomes, including reduced quality-adjusted life years, disease-free survival, and, in the worst-case scenario, surgery cancellations, which also negatively affect hospital finances [9–11].

Hence, protecting at-risk healthcare workers (HCWs) is critical, and knowing that new pandemics beyond COVID-19 will inevitably emerge, a systematic decision support tool to protect HCWs during a pandemic is needed. Over the last 2 years, various staffing strategies have been tested or implemented in hospitals to protect HCWs, and we identified three simulation-based studies [12]. The first study reported that longer shifts and avoiding staggering rotations of HCW shifts could reduce infection among HCWs [13]. The second study observed that the longer shifts with continuous breaks significantly reduced HCW shortage, and the effect progressively increased as the probability of infection increased [14]. Finally, the third study implemented a staffing policy where neurosurgery residents were divided into two teams and alternated weekly with minimal contact, and it showed that the teaming approach minimised the risk of exposure and provided the needed rest for residents [15].

Although prior studies report the effectiveness of various strategies in reducing infections among HCWs and their unavailability, these have been restricted to a single, small facility and do not account for how individual system factors interact with each other and impact the infection spread. By including factors such as vaccination rate among HCWs, infection transmission rates at each facility, the number of patient–HCW and HCW–HCW interactions, and patient census in the hospital, we developed simulation models using the agent-based modelling (ABM) approach to gain in-depth insights into disease spread and identify the best staffing policy that minimises infection spread and unavailability among the perioperative team at Prisma Health, SC.

## Methods

### Data, assumptions, and model development

Data used in this study regarding bed capacity, locations, and perioperative HCWs required were derived from Prisma Health Upstate, which did not include any identifiers. The study was provided with an institutional review board (IRB) exemption by the Prisma Health IRB. The rest of the data used in the study were collected from publicly available epidemiologic data about COVID-19. We consider three different locations of Prisma Health Upstate and three different types of HCWs (anaesthesiologists, anaesthetists, and nurses) who are part of the perioperative team. Among these, locations 1 and 2 are regular facilities receiving patients of all types, whereas location 3 was transitioned to treat only COVID-19 patients because of the surge experienced. There are 853 beds available for patient care in total (facility 1: 700; facility 2: 108; facility 3: 45), including inpatient beds and operating rooms (ORs). In our model, we did not specifically focus on the OR workflow. Instead, we focused on the inpatient beds and interactions out-of-the-OR activity (recovery room, workstation, etc.). The primary reason for this was that we assume that clinicians are masked and protected in the OR, whereas they might not be in the recovery room and workstation. We consider the number of interactions between each patient and each HCW type as a key factor in our ABM, which allows us to capture the impact of HCW availability on their workload in terms of patient interactions and the likelihood of getting infected. In our model, although we use a fixed transmission probability per interaction, the probability of an HCW getting infected is not static. We consider it as a function of total HCWs available to work, patient volume, and the average number of interactions with patients, according to the following formula: 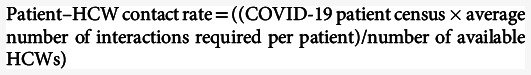


Here the COVID-19 patient census would vary based on the scenario under consideration (discussed in the next paragraph). The average number of interactions required per patient is based on the HCW type, where we assume nurses have more contact with patients on average than anaesthetists. The number of HCWs represents the healthy workforce of each HCW available in the hospital. The motivation to use this equation here is to account for the varying HCW workload during a shortage of workforce or surge in COVID-19 patients without detailed modelling of the complex workflow, which is significantly different for an OR versus an inpatient bed. Due to the lack of detailed data on the number of interactions required per patient with HCWs and the characterisation of interactions among HCWs themselves in their workspace, we set these numbers in our experiments based on expert opinions from HCWs in the Prisma Health Department of Anesthesiology (see Table 1). Here the number of interactions follows the CDC’s guidelines for close contact, which is less than 6 feet away from a person for a total of 15 minutes or more. The interactions between anaesthesiologists represent their interactions in the recovery room, workstation, and so forth, and not while caring for patients. For nurses, their interactions represent their interactions in workstations and while passing by between inpatient beds. The number of interactions between anaesthetists represents those outside the OR. While it could be possible that there might be no interaction between each HCW, we assume they could interact while passing by inpatient beds, workstations, lockers, or while passing by ORs. For the data on testing frequency and quarantine period, we followed the policies and practices at Prisma Health during January 2022. The data pertinent to COVID-19 transmission probabilities, incubation time, presymptomatic time, asymptomatic and symptomatic probability, recovery period, and mortality rate were obtained from publicly available Centers for Disease Control and Prevention (CDC) guidelines and literature from February 2022 [16]. For HCWs returning to work after mandatory quarantine, the possibility of reinfection was considered since multiple studies reported such cases [17, 18]. Finally, an infection possibility after vaccination, as represented in Table 1, was also considered, as prior studies observed that no vaccination provided 100% protection against COVID-19.

**Table 1.** Model parameters

Parameter	Value
HCW-to-HCW transmission rate	4.00%
Number of interactions between anaesthesiologists	3 per hour
Number of interactions between nurses	3 per hour
Number of interactions between anaesthetists	1 per hour
Number of interactions between each HCW type	1 per hour
Patient transmission rate	0.04 or 0.004%
Interactions with anaesthesiologists per patient	2
Interactions with nurses and anaesthetists per patient	3
Incubation period	Triangular (2,4,12) days
Presymptomatic period	0–2 days
Asymptomatic probability	40%
Workforce testing frequency	1 per week
Quarantine period	5/10/14 days based on vaccination
Mortality rate	1.8%
Vaccinated	0 or 50 or 75 or 90%
Reinfection probability	0.0004%
Transmission probability (after vaccination)	12.5% of transmission rate (0.125 × transmission rate)

Abbreviation: HCW, healthcare worker.

Although our model does not explicitly consider factors outside of the hospital, to replicate the population dynamics, we consider three different scenarios for patient census represented by the percentage of hospital bed occupancy by COVID-19 patients at each facility: (i) low patient census (5–10%), (ii) medium patient census (20–25%) and (iii) high patient census (>50%). Additionally, we also consider two infection transmission rates: low and high transmission scenarios. Finally, we consider four different scenarios where 0%, 50%, 75%, and 90% of HCWs are vaccinated to evaluate the impact of vaccination rates. Although these combinations of factors (patient census, transmission rates, vaccination rates) do not come from actual scenarios at the partner hospital, the research team aimed to model and investigate these different scenarios to capture different population dynamics stages (early stage, peak infection, and recovery) for COVID-19 or similar pandemic. Table 1 summarises the key input parameters used for our model.

**Figure 1.**
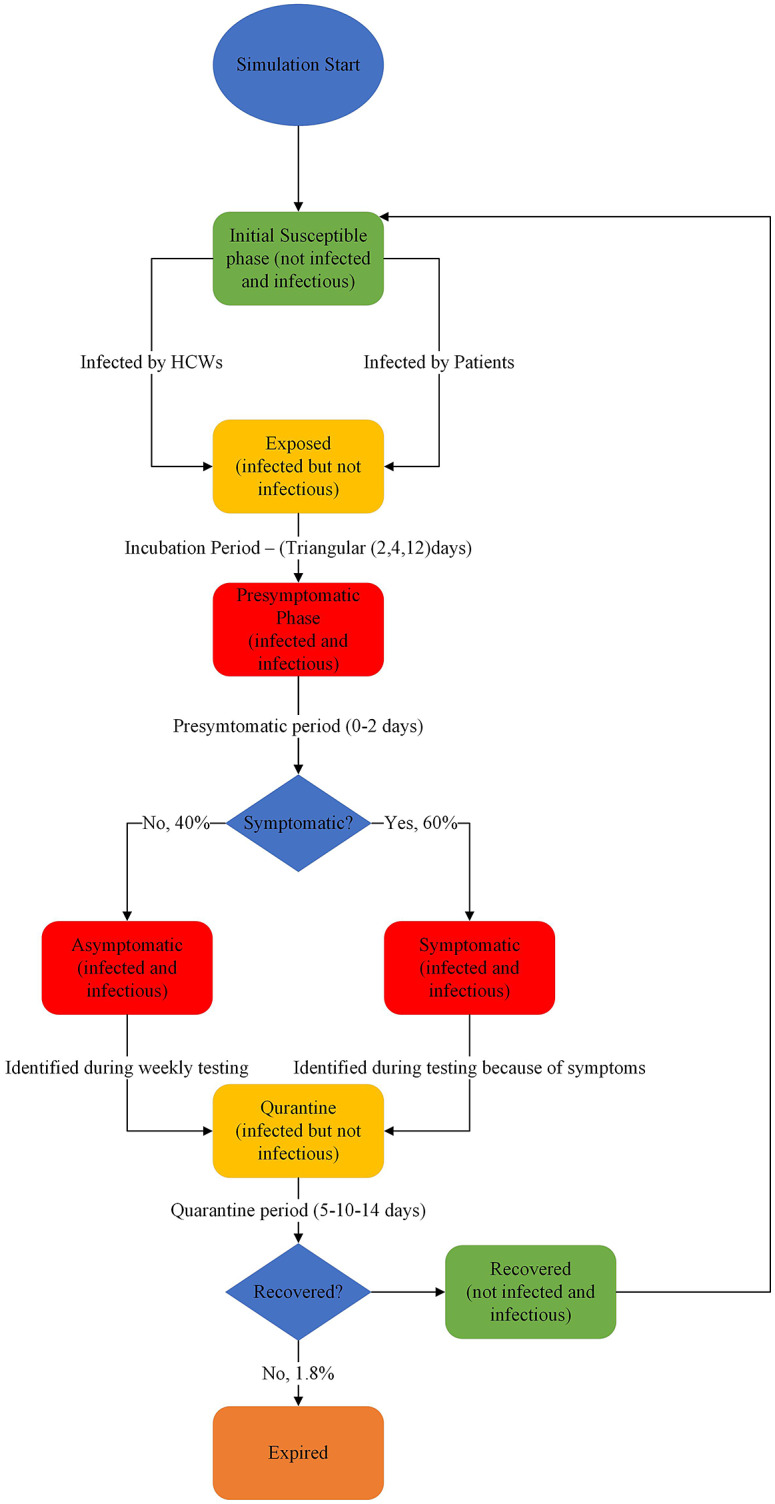
Healthcare worker (HCW) process flow map.

We developed simulation models using ABM where each HCW is considered a unique agent with specific parameters, attributes, and rules by which they function in a hospital. Additionally, using the ABM approach allowed us to track the current state (in terms of Susceptible–Exposed–Infected–Recovered) of each HCW as the simulation progressed. Before initiating the simulation, each agent is first scheduled to work at a specific hospital location for a week. Based on the policy under consideration, each HCW is assigned a list of HCWs with whom they can potentially interact within the hospital. We considered all HCWs as the susceptible population at the beginning of the simulation and assumed they could be infected by either patients or other HCWs. If infected, instead of going directly into the state of being infectious, the HCW moves on first to the exposed state, where they stay for a certain period of time (referred to as the incubation period). In this exposed state, a provider is infected but not infectious, meaning they cannot spread the disease. Following the exposed state, they move on to the so-called presymptomatic phase, where they do not present any symptoms but are infectious, meaning they can potentially infect other HCWs. The presymptomatic phase is followed by a symptomatic or asymptomatic infectious state where symptomatic HCWs start exhibiting COVID-19 symptoms as opposed to asymptomatic HCWs who do not. Symptomatic HCWs are tested immediately and follow appropriate quarantine protocols. Asymptomatic HCWs continue to spread the infection to other HCWs unless they test positive during routine weekly testing, after which they follow quarantine protocols. Following quarantine procedures, there is a small probability that an agent might expire, but most of them would recover and enter the work system where they could be reinfected. The different stages an HCW may progress through during the simulation can be seen in Fig. 1.

### Staffing policies and simulated cases

To identify the best staffing strategy that could minimise the number of infections and unavailability among HCWs, we compared six staffing policies under different scenarios of patient volume, vaccination status, and infection transmission rates. Based on expert opinions from the Department of Anesthesiology at Prisma Health Upstate, we used the weekly availability of HCWs and the total number of HCWs infected as the two primary outcomes to compare various staffing policies. Below are the details of staffing policies we investigate.

#### Policy 1: Inter-hospital mixing (baseline policy/current practice)

This policy corresponds to the current practice in the partner health system, where an HCW is allowed to work in any facility. Specifically, an HCW is assumed to have the option to switch facilities and/or groups every week but will work at the same facility each week. This policy allows the highest flexibility in staffing and scheduling. In our simulation model, the assignments of HCWs to facilities and groups are generated randomly.

#### Policy 2: Inter-group mixing

In this policy, we first divide HCWs into groups and restrict the HCWs’ interactions by restricting their shift options only to those available within a facility. Here an HCW is allowed to switch groups within the same facility but cannot sign up for a shift in a different facility.

#### Policy 3: No mixing

In this policy, we further restrict the interactions among HCWs by segregating them into predefined groups within a single facility. They can only bid for a particular shift, and they stay with the same team throughout the simulation study horizon.

#### Policies 4–6: Rotating schedule

With these policies, we reduce the number of HCWs present in the hospital by implementing a rotation schedule. Specifically, at any given time, we assign 67% of the HCWs to work and the other 33% to stay at home, and these groups are rotated every 2 weeks. We combine this rotating policy with the aforementioned three policies – inter-hospital mixing, inter-group mixing, and no mixing – to obtain policies 4–6, respectively.

All of these policies were developed based on discussions with the providers at Prisma Health Upstate to ensure their realism and generality such that they can be adopted into any health system with multiple facilities. Specifically, based on discussions with expert clinicians working in perioperative settings, we used 7.5% of bed capacity as the low capacity, 22.5% as medium capacity, and 65% as a high capacity when these beds (inpatient beds + OR) were occupied by COVID-19 patients. We evaluate the performance of different policies under multiple scenarios where we vary patient census, vaccination status, and infection transmission probabilities. As mentioned earlier, these scenarios are not actual scenarios observed in the partner hospital. Instead, we consider various combinations of these factors as they allow us to differentiate between different types and sizes of healthcare facilities, reflect the impact of state/local policies, and model both high- and low-risk geographical locations. Specifically, we tested the six staffing policies as below:

- Case 1: Low patient census and high patient transmission rate
- Case 2: Medium patient census and high patient transmission rate
- Case 3: High patient census and high patient transmission rate
- Case 4: Low patient census and low patient transmission rate
- Case 5: Medium patient census and low patient transmission rate
- Case 6: High patient census and low patient transmission rate.

Two hundred replications of simulations were run for each combination of the parameters such that the reported metrics for the total number of infected HCWs were with a 99% confidence interval of ±.01. A one-way ANOVA was utilised to compare if the total number of HCWs infected under each policy was statistically significantly different. In case of significant differences for the ANOVA, it was followed with a Tukey post-hoc to identify the groups that varied statistically. For both statistical tests, an *α* = 0.01 was used.

## Results

In this section, we summarise the performances of the six staffing policies presented above. Given that there are a variety of combinations, we present results in such a way that cases 1–3 and 4–6 are averaged and represented in two separate tables for each vaccination rate. In each schedule, the relative ratio of HCWs was 64.3% nurses, 27.46% nurse anaesthetists, and 8.1% anaesthesiologists.

**Figure 2.**
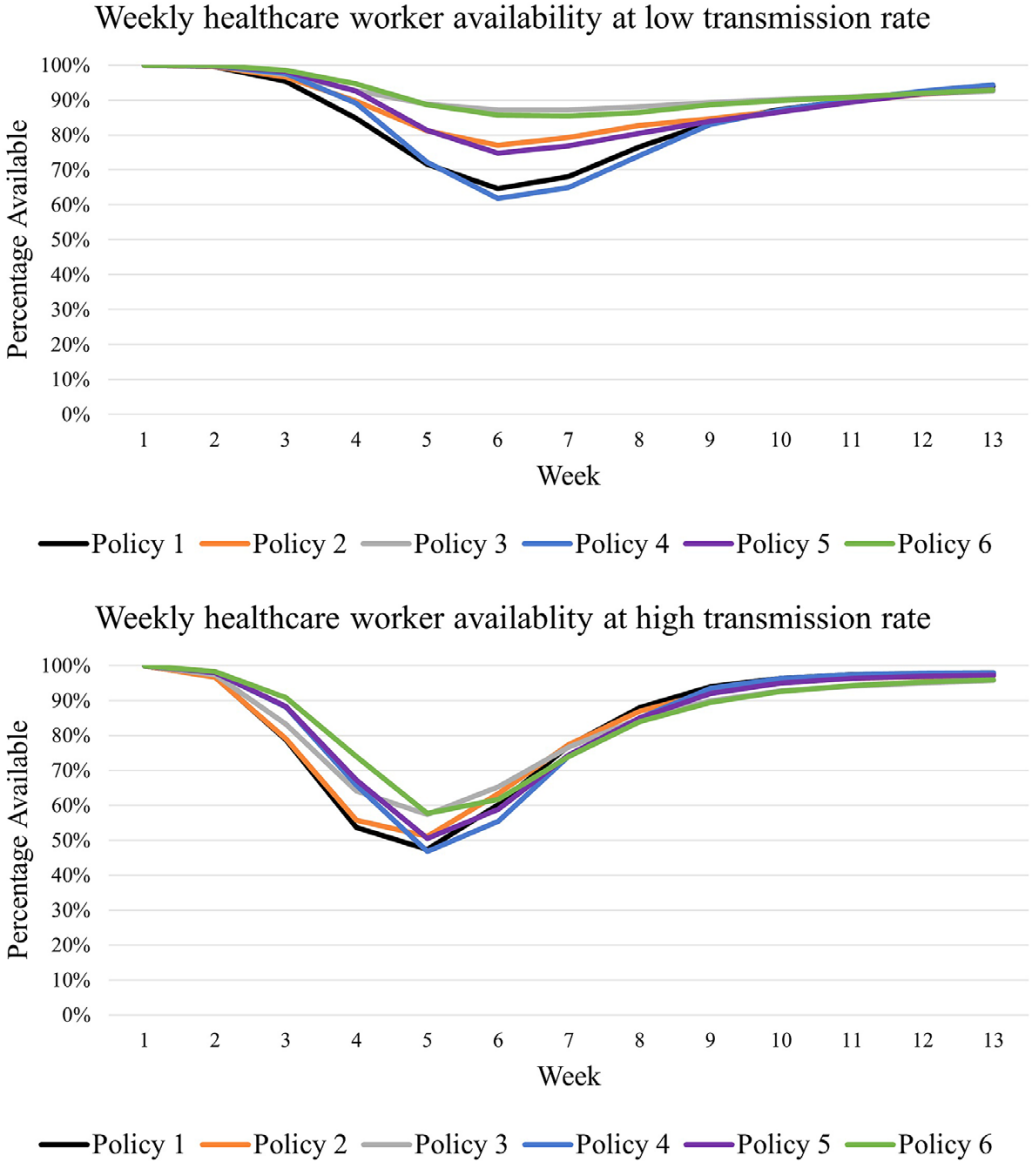
Weekly healthcare worker (HCW) availability at low and high transmission rates with 0% vaccination.

On investigating the scenario with a vaccination rate of 0%, which corresponds to the early phase of the pandemic when no vaccines are available, the model predictions showed a significant difference (*p*-value <0.01) between the six policies in terms of the total number of HCWs infected when patient transmission rates were lower (cases 4–6) irrespective of the patient census, as represented in Table 2. Specifically, no mixing (policy 3) and rotation–no mixing (policy 6) policies were significantly (*p*-value <0.01) better than the inter-group and inter-hospital mixing policies as well as their rotation versions (policies 1, 2, 4, and 5). Additionally, simulated findings showed that inter-group mixing (policy 2) and its rotation version (policy 5) were significantly (*p*-value = 0.01) better than inter-hospital mixing and its rotation version (policies 1 and 4). Regarding the peak weekly unavailability of HCWs, model outcomes were similar, where no mixing (policy 3) and its rotation version (policy 6) outperformed other policies (policies 1, 2, 4, and 5) by improving weekly HCW availability by as much as 22%, as shown in Table 3. Further, inter-group mixing (policy 2) and its rotation version (policy 5) outperformed inter-hospital mixing and its rotation version (policies 1 and 4) by improving weekly HCW availability by 12%. Fig. 2 represents the weekly availability of HCWs at a low transmission rate. Finally, comparing the rotation policies (policies 4–6) to the respective restriction policies (policies 1–3), the model predictions did not vary significantly in terms of weekly provider unavailability and the total number of HCWs infected.

**Table 2.** Total healthcare workers infected over 90 days at four vaccination rates

Vaccination (%)	Cases	Policy 1	Policy 2	Policy 3	Policy 4	Policy 5	Policy 6
0	1	98.7 ± 0.10	97.2 ± 0.90	91.8 ± 0.91	98.0 ± 0.22	97.1 ± 0.80	91.6 ± 0.71
	2	98.6 ± 0.11	98.7 ± 0.32	98.4 ± 0.62	98.2 ± 0.70	98.7 ± 0.39	98.1 ± 0.56
	3	98.9 ± 0.09	99.0 ± 0.11	98.7 ± 0.41	98.9 ± 0.01	99.0 ± 0.00	99.0 ± 0.00
	4	83.2 ± 1.05	50.1 ± 0.91	26.1 ± 1.10	83.0 ± 1.10	50.1 ± 0.87	26.4 ± 0.44
	5	98.0 ± 0.12	82.7 ± 0.42	59.4 ± 0.01	98.3 ± 0.62	81.7 ± 0.66	59.0 ± 0.14
	6	98.8 ± 0.90	95.5 ± 0.19	83.3 ± 0.45	98.9 ± 0.87	94.7 ± 0.70	80.9 ± 1.01
50	1	47.9 ± 1.22	46.0 ± 0.98	40.6 ± 0.90	48.2 ± 1.01	45.8 ± 0.71	41.0 ± 0.88
	2	48.0 ± 0.34	48.1 ± 0.55	47.9 ± 0.81	48.5 ± 0.15	48.3 ± 0.11	48.0 ± 0.56
	3	49.1 ± 0.75	49.0 ± 0.43	48.3 ± 0.33	49.4 ± 0.12	48.6 ± 0.10	48.3 ± 0.31
	4	29.0 ± 0.33	13.3 ± 0.45	7.9 ± 0.24	28.7 ± 0.80	13.0 ± 1.01	8.1 ± 0.94
	5	45.9 ± 0.54	28.5 ± 0.62	20.1 ± 0.12	45.8 ± 0.79	28.4 ± 0.90	20.2 ± 0.56
	6	48.7 ± 0.32	43.0 ± 0.74	36.7 ± 0.85	48.9 ± 0.62	43.0 ± 0.55	36.6 ± 0.41
75	1	23.0 ± 0.65	21.1 ± 0.40	19.5 ± 0.70	22.8 ± 0.58	21.0 ± 0.90	19.5 ± 0.11
	2	24.2 ± 0.45	23.8 ± 0.87	24.0 ± 0.09	24.0 ± 0.80	23.8 ± 0.94	23.7 ± 0.16
	3	25.5 ± 0.33	25.0 ± 0.61	25.1 ± 0.33	25.0 ± 0.23	25.1 ± 0.77	24.4 ± 0.29
	4	8.0 ± 0.85	6.7 ± 0.89	4.0 ± 0.80	8.0 ± 0.67	6.5 ± 0.22	3.9 ± 0.75
	5	19.2 ± 1.13	13.3 ± 0.81	9.3 ± 0.47	19.3 ± 0.90	13.2 ± 0.34	9.2 ± 0.40
	6	24.3 ± 0.72	20.5 ± 1.22	16.3 ± 0.29	24.3 ± 0.73	20.8 ± 0.32	16.3 ± 0.87
90	1	7.2 ± 0.17	7.2 ± 0.87	5.8 ± 0.67	7.2 ± 0.30	7.1 ± 0.91	5.8 ± 0.62
	2	9.4 ± 0.30	9.4 ± 0.04	8.6 ± 0.20	9.1 ± 0.69	9.1 ± 0.80	8.0 ± 0.99
	3	10.1 ± 0.39	10.1 ± 0.65	10.0 ± 0.89	10.3 ± 0.20	10.0 ± 0.92	9.9 ± 0.41
	4	1.9 ± 0.66	1.6 ± 0.57	1.4 ± 0.42	2.1 ± 0.46	1.6 ± 0.85	1.5 ± 0.98
	5	4.3 ± 0.75	3.5 ± 0.31	3.1 ± 0.51	4.0 ± 0.90	3.2 ± 0.72	3.0 ± 0.57
	6	8.1 ± 0.83	7.7 ± 0.49	7.0 ± 1.10	8.1 ± 1.10	7.5 ± 0.56	6.9 ± 0.75

**Table 3.** Confidence intervals for a few policies (most significant) where the total of healthcare workers were infected over 90 days at four vaccination rates

Vaccination (%)	Patient transmission rates	Policies with significant differences
0	Low transmission	P3–P1, CI [−57.2, −56.1]
		P3–P2, CI [−24.3, −23.0]
		P2–P1, CI [−33.2, −32.7]
		P6–P4, CI [−56.8, −55.7]
		P6–P5, CI [−24.1, −23.2]
		P5–P4, CI [−33.1, −32.0]
0	High transmission	No significant differences
50	Low transmission	P3–P1, CI [−25.5, −24.8]
		P3–P2, CI [−7.7, −6.9]
		P2–P1, CI [−17.2, −16.5]
		P6–P4, CI [−25.0, −14.0]
		P6–P5, CI [−7.1, −6.5]
		P5–P4, CI [−16.6, −15.9]
50	High transmission	No significant differences
		Except for low census
75	Low transmission	P3–P1, CI [−8.0, −7.1]
		P3–P2, CI [−4.5, −3.2]
		P6–P4, CI [−7.8, −7.0]
		P6–P5, CI [−3.9, −3.2]
75	High transmission	No significant differences
90	Low transmission	No significant differences
90	High transmission	No significant differences

At the same vaccination rate (0%), when patient transmission rates were higher (cases 1–3), the total number of HCWs infected over the 90 days did not vary significantly (*p*-value = 0.38) between the six staffing policies, as shown in Table 2, as eventually all HCWs got infected. However, on comparing the weekly unavailability of HCWs under each policy, no mixing (policy 3) and its rotation version (policy 6) outperformed other policies (policies 1, 2, 4, and 5) by improving weekly HCW availability by as much as 11%, as shown in Table 3. Although not much, the inter-group mixing (policy 2) and its rotation version (policy 5) outperformed the inter-hospital mixing and its rotation version (policies 1 and 4) by improving weekly HCW availability by 5%. Fig. 2 represents the weekly availability of HCWs at a high transmission rate, where it can be seen that HCW unavailability peaks when transmission rates tend to be higher, representing an increased likelihood of infection spread. Finally, for the weekly unavailability of HCWs, irrespective of patient transmission rates, the rotation policies (policies 4–6) could delay the peak of HCW unavailability, reduce the duration of peak unavailability and reduce the peak compared to respective restriction policies (policies 1–3) as shown in Table 3.

**Figure 3.**
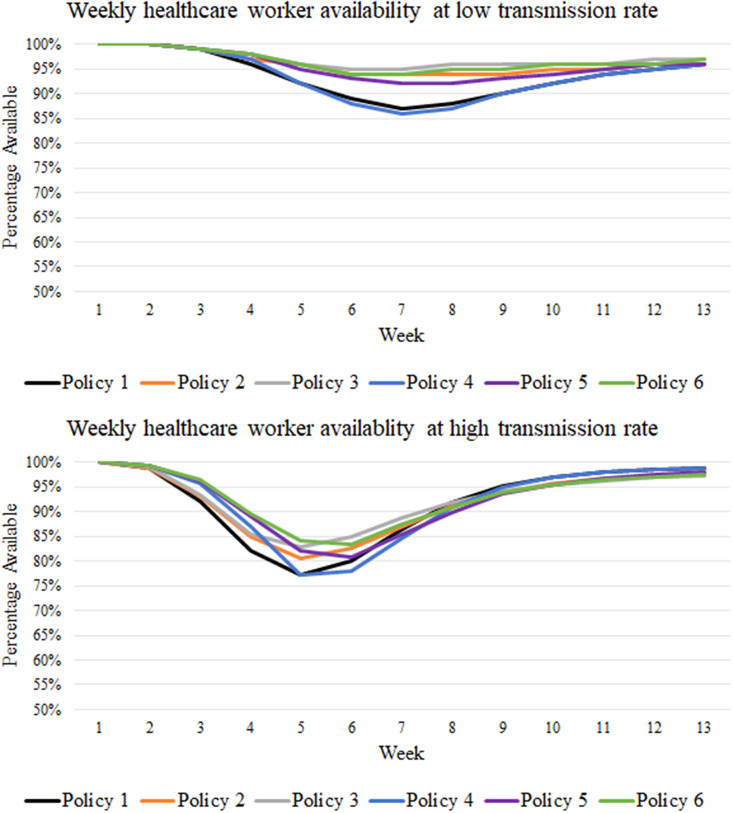
Weekly healthcare worker (HCW) availability at low and high transmission rates with a 50% vaccination.

Next, we investigate the 50% vaccination scenario, and similar to the 0% vaccination scenario, the total number of HCWs getting infected at low patient transmission rates (cases 4–6) was significantly (*p*-value = 0.01) different across the six policies. Specifically, no mixing (policy 3) and rotation–no mixing (policy 6) policies significantly (*p*-value <0.01) reduced the total number of HCWs infected when compared to the inter-group, inter-hospital, and rotation versions of these policies (policies 1, 2, 4, and 5), as represented in Table 2. Likewise, inter-group mixing (policy 2) and its rotation version (policy 5) performed significantly (*p*-value = 0.03) better than inter-hospital mixing and its rotation version (policies 1 and 4). In terms of the peak weekly unavailability of HCWs, when patient transmission rates were low, no mixing (policy 3) and its rotation version (policy 6) were the best but outperformed inter-hospital mixing and its rotation version (policies 1 and 4) by improving the peak weekly HCW availability by as much as 8%, as represented in Table 4. Further, from Table 4, it can be observed that inter-group mixing (policy 2) and its rotation version (policy 5) outperformed inter-hospital mixing and its rotation version (policies 1 and 4) by increasing the peak weekly HCW availability by 7%. Fig. 3 represents the weekly availability of HCWs at a low transmission rate.

**Table 4.** Average weekly healthcare worker availability for low and high transmission rates at 0% vaccination

Transmission rate	Week	Policy 1 (%)	Policy 2 (%)	Policy 3 (%)	Policy 4 (%)	Policy 5 (%)	Policy 6 (%)
Low transmission rate	1	100	100	100	100	100	100
	2	100	100	100	100	100	100
	3	95	97	97	98	98	99
	4	85	90	93	89	93	95
	5	72	81	89	72	81	89
	6	65	77	87	62	75	86
	7	68	79	87	65	77	85
	8	77	83	88	74	80	86
	9	83	85	89	83	84	89
	10	87	87	90	87	87	90
	11	90	90	91	90	89	91
	12	92	92	92	93	92	92
	13	94	93	93	94	93	93
High transmission rate	1	100	100	100	100	100	100
	2	97	97	97	98	98	98
	3	79	79	83	88	88	91
	4	54	56	64	66	67	74
	5	47	51	57	47	51	58
	6	60	63	65	55	59	62
	7	77	77	77	74	74	74
	8	88	87	85	85	85	84
	9	94	92	90	94	92	90
	10	96	95	93	96	95	93
	11	97	96	94	97	96	94
	12	98	97	95	98	97	95
	13	98	97	96	98	97	96

At high transmission rates (cases 1–3), there were no significant differences in the total number of HCWs who were infected, as represented in Table 2, except for the low patient census scenario where no mixing (policy 3) and its rotation version (policy 6) significantly (*p*-value = 0.02) outperformed inter-hospital mixing and its rotation version (policies 1 and 4). However, model predictions showed that no mixing (policy 3) and its rotation version (policy 6) outperformed other policies (policies 1 and 4) by improving weekly HCW availability by as much as 7%, as shown in Table 4. Further, inter-group mixing (policy 2) and its rotation version (policy 5) slightly outperformed inter-hospital mixing and its rotation version (policies 1 and 4) by improving weekly HCW availability by 4%. Fig. 3 represents the weekly availability of HCWs at a high transmission rate, where it can be noticed that as vaccination rates start to increase, the distinction between no mixing (policies 3 and 6) and inter-group mixing policies (policies 2 and 5) reduces in terms of lowering weekly HCW unavailability. However, these still outperform inter-hospital mixing policies (policies 1 and 4). Finally, on comparing the model predictions, we observed that the rotation policies (policies 4–6) could delay the peak of HCW unavailability, reduce the duration of peak unavailability and reduce the peak compared to respective restriction policies (policies 1–3) irrespective of patient transmission rates.

It is evident that both restriction and rotation policies can help reduce the total number of HCWs getting infected and the weekly unavailability of HCWs when vaccination rates among providers are no more than 50%. To identify if these policies can have the same impact as when vaccination rates increase, we consider the scenario where 75% of HCWs were vaccinated. On comparing the model predictions for the six staffing policies, we observed that restriction and rotation policies did not add a significant value in terms of reducing weekly HCW unavailability irrespective of patient transmission rates, as represented in Supplementary Figure S1 and Supplementary Table S1. However, when patient transmission rates were lower (cases 4–6), no mixing (policy 3) and rotation–no mixing (policy 6) were significantly (*p*-value = 0.03) better than inter-hospital mixing, inter-group mixing, and their rotation versions (policies 1, 2, 4 and 5) in terms of reducing the total number of HCWs infected, as presented in Table 2. Here inter-group mixing and its rotation version (policies 2 and 5) did not vary significantly (*p*-value = 0.07) from inter-hospital mixing and its rotation version (policies 1 and 4). Although the benefits of restriction and rotation policies have started to diminish, it is apparent that they can still add value (Table 2).

**Table 5.** Average weekly healthcare worker availability for low and high transmission rates at 50% vaccination

Transmission rate	Week	Policy 1 (%)	Policy 2 (%)	Policy 3 (%)	Policy 4 (%)	Policy 5 (%)	Policy 6 (%)
Low transmission rate	1	100	100	100	100	100	100
	2	100	100	100	100	100	100
	3	99	99	99	99	99	99
	4	96	97	98	97	98	98
	5	92	96	96	92	95	96
	6	89	94	95	88	93	94
	7	87	94	95	86	92	94
	8	88	94	96	87	92	95
	9	90	94	96	90	93	95
	10	92	95	96	92	94	96
	11	94	95	96	94	95	96
	12	95	96	97	95	96	96
	13	96	96	97	96	96	97
High transmission rate	1	100	100	100	100	100	100
	2	99	99	99	99	99	99
	3	92	93	93	96	96	96
	4	82	85	85	87	89	89
	5	77	81	83	77	82	84
	6	80	83	85	78	81	83
	7	86	87	89	85	86	87
	8	92	91	92	91	90	91
	9	95	94	94	95	94	94
	10	97	96	95	97	96	95
	11	98	97	96	98	97	96
	12	99	97	97	99	97	97
	13	99	98	97	99	98	97

Finally, to understand if these restriction and rotation policies can still add value, we consider an extreme scenario where 90% of HCWs are vaccinated. Regardless of patient census and patient transmission rates, the model predictions did not show a significant (*p*-value = 0.12) difference in the total number of HCWs infected, as shown in Table 2. Similarly, for the weekly unavailability of HCWs, as observed under 75% vaccination, there was no major difference as the maximum unavailability was only 3% for a week, as shown in Supplementary Figure S2 and Supplementary Table S2. The findings suggest that restriction policies and rotation policies do not add significant value when vaccination rates are high and chances of infection after vaccination are lower. Depending on patient transmission rates, restriction, and rotation policies can still be beneficial by flattening the curve of HCW unavailability and delaying the peak compared to the baseline policy.

## Discussion

In this research, we explored the impact of segregating and rotating HCW staffing shifts in a large health system having multiple locations to address staff unavailability due to infections during the COVID-19 pandemic and, moreover, prepare for a similar future potential pandemic. Although prior studies have reported that rotation schedules and continuous breaks could improve HCW availability and reduce rates of infection in a single location, none of these studies has considered varying patient census, transmission rates, vaccination rates and multiple hospital locations [13–15]. We investigated rotation policies along with novel policies that segregate HCWs into smaller groups within a facility and restricted them to a single facility. By simulating various scenarios with different factors, including patient census, transmission rates, vaccination rates at multiple hospital locations, and other COVID-19 data, we observed that risk-adjusted staffing could reduce both total HCW unavailability and most severe staff shortages. Specifically, findings from our simulation model suggest that segregating HCWs into smaller groups within a facility, restricting them to a single facility, and rotating (alternating) HCW shifts can significantly reduce COVID-19 exposure and infection spread, thereby reducing HCW shortage. The findings about rotation schedules align well with prior single-site studies; in addition, we also provided empirical performances of two additional policies (segregation within a facility and restricting to a single facility) through our simulation models, which other health systems can implement to improve HCW availability.

Moreover, the restricted team mixing and rotating staffing schedules always performed similar to or better than the current practice, and our model predictions showed that the value of rotating and restriction policies diminished as vaccination rates among HCWs increased. Specifically, when 50% or lesser of HCWs were vaccinated, the restriction and rotation policies significantly reduced both the weekly unavailability of HCWs and the total number of HCWs getting infected by as much as 22% and 38% compared to the current practice (*p*-value <0.01). However, when 75% of HCWs were vaccinated, these policies were only helpful in reducing the total number of HCWs getting infected (*p*-value = 0.03), and when 90% of HCWs were vaccinated, these restrictions did not have any significant effect (*p*-value = 0.09). These findings from our simulation model indicate that the vaccination status of HCW population should also be considered as a key variable while developing staffing strategies and schedules during a pandemic.

While our study focused on anaesthesiologists, anaesthetists, and nurses in the perioperative population, these findings might be generalisable to other departments with a high risk of infectivity and HCW unavailability. Similarly, our findings are based on COVID-19 data, but we believe these findings can be helpful in preparing hospitals, especially larger health systems with multiple locations, against future pandemics or similar viral infections. Additionally, the input parameters of the simulation model can be easily customised for different infectious disease scenarios, allowing its use in other pandemics or conditions where HCWs could be unavailable. Moreover, these models can be shared with other health systems where they can manipulate facility-specific numbers (beds, locations, number of HCWs, etc.) to match their needs.

Finally, we would like to point out a few limitations of this study. First, the analysis and results are based on simulated findings as opposed to empirical results. However, our simulated results are reported with a 99% confidence interval. Another limitation is that we assume that each patient, on average, comes in contact with a provider a certain number of times, and providers interact with each other at a particular rate. Although these assumptions are based on expert opinions from anaesthesiologists working in the partner hospital, including the second author, we recognise the fact that the number of actual interactions could be higher in the OR when HCWs could be in close contact most of the time, or lower while providing care on inpatient beds, depending on the scenario. However, to reduce the complexity of modelling these different workflows, we decided to use the average, as we aimed to compare various staffing policies (flexible vs. restricted) during various stages of a pandemic (early, medium, and late) without changing any workflow/processes. In the future, the model could be updated to incorporate a detailed workflow. Another limitation is associated with modelling and replicating the partner hospital’s activities. While physicians were involved throughout the model development process to replicate the actions, we acknowledge that certain assumptions (interactions) and simplifications of complex workflow in the model could limit the ability to replicate the activities at the partner hospital completely. Comparing the two studies that investigated unscheduled absences among HCWs in a similar setting, which reported an increase in the odds of unscheduled absences with an increase in asymptomatic COVID-19 patients, while our findings align with the observation of increased unavailability among HCWs, our numbers look comparatively inflated [19, 20]. One of the primary reasons for the discrepancy in findings is that different performance metrics are used by the two studies. Moreover, it can be observed from our results that the inflated numbers are reported for the worst-case scenario (high COVID-19 patient census and low vaccination); during other scenarios (high vaccination and low patient census), our findings are similar to those reported in the literature. Finally, the time until provider availability after infection is based on recovery time and the isolation guidelines from the CDC, but we recognise that some hospitals may have different practices, and these durations might vary.

## Data Availability

All the data used in the model are provided in the manuscript, and the corresponding author can answer any additional questions.

## Supplementary material

For supplementary material accompanying this paper visit https://doi.org/10.1017/S0950268823000511. click here to view supplementary material
