# Supplementary material for: Risk-adjusted policies to minimise perioperative staffing shortages during a pandemic: An agent-based simulation study
Source: Epidemiol Infect. 2023 Apr 3;151:e66. doi: 10.1017/S0950268823000511 (PMC10154643; doi:10.1017/S0950268823000511)
Supplement: Supplementary file 1 [file S0950268823000511sup001.docx]

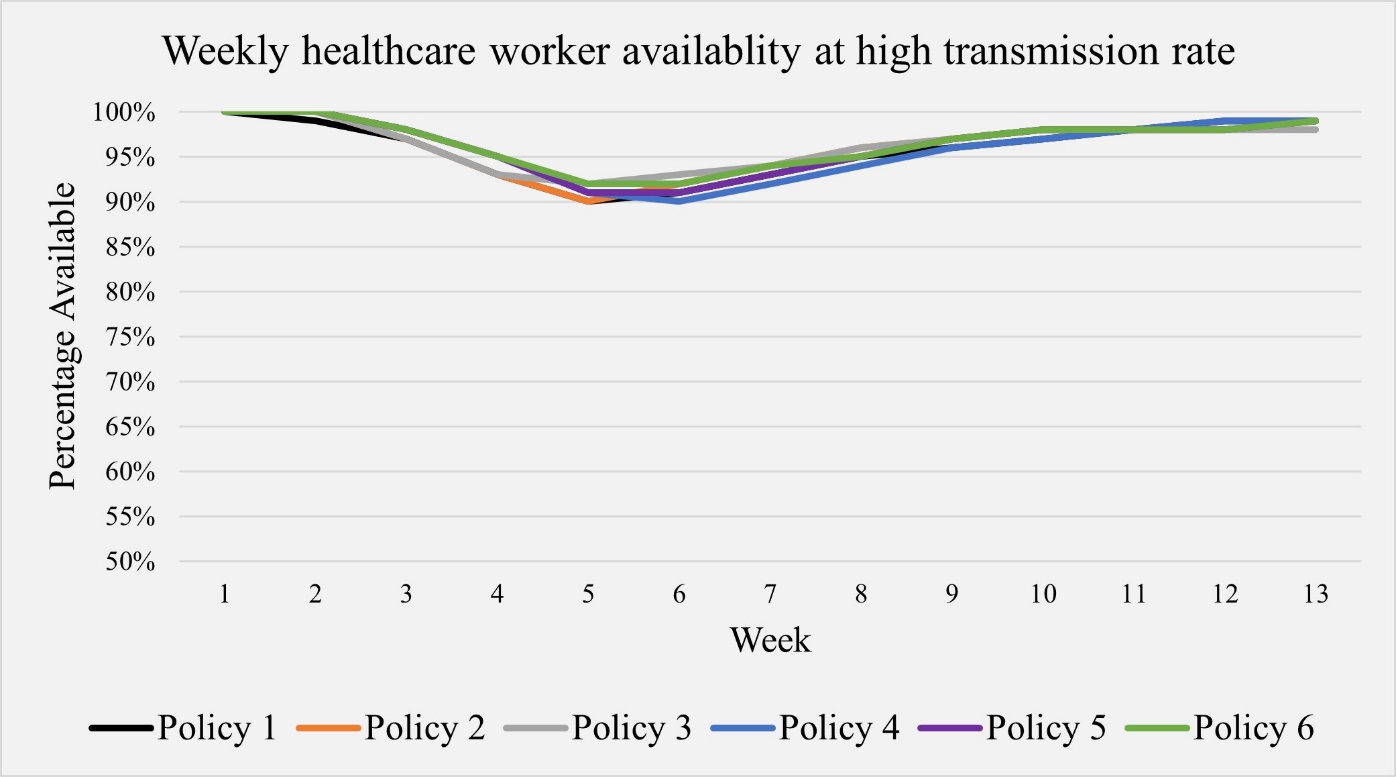


**Supplemental Figure S1.** Weekly healthcare worker (HCW) availability at a high transmission rate with a 75% vaccination.


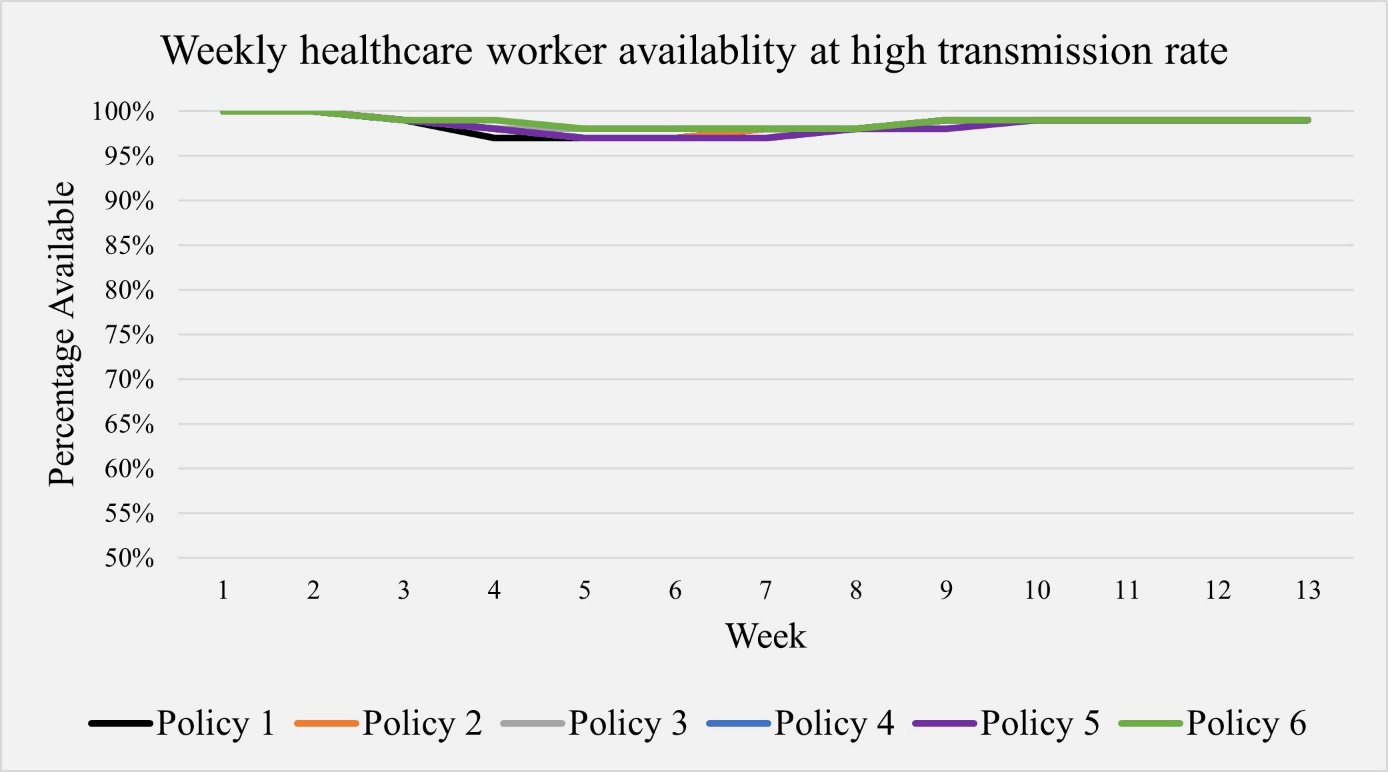


**Supplemental Figure S2.** Weekly healthcare worker (HCW) availability at a high transmission rate with a 90% vaccination.

**Supplemental** **Table S1.** Average weekly healthcare worker (HCW) availability for low and high transmission rates with a 75% vaccination.

| **Transmission**  **Rate** | **Week** | **Policy 1** | **Policy 2** | **Policy 3** | **Policy 4** | **Policy 5** | **Policy 6** |
| --- | --- | --- | --- | --- | --- | --- | --- |
| Low Transmission  Rate | 1 | 100% | 100% | 100% | 100% | 100% | 100% |
|  | 2 | 100% | 100% | 100% | 100% | 100% | 100% |
|  | 3 | 100% | 100% | 100% | 100% | 100% | 100% |
|  | 4 | 99% | 99% | 99% | 99% | 99% | 99% |
|  | 5 | 98% | 98% | 98% | 98% | 97% | 98% |
|  | 6 | 97% | 97% | 98% | 96% | 97% | 98% |
|  | 7 | 96% | 97% | 98% | 96% | 96% | 97% |
|  | 8 | 96% | 97% | 98% | 95% | 96% | 98% |
|  | 9 | 96% | 97% | 98% | 96% | 97% | 98% |
|  | 10 | 96% | 97% | 98% | 96% | 97% | 98% |
|  | 11 | 97% | 98% | 98% | 97% | 97% | 98% |
|  | 12 | 97% | 98% | 98% | 97% | 98% | 98% |
|  | 13 | 98% | 98% | 98% | 98% | 98% | 98% |
| High Transmission  Rate | 1 | 100% | 100% | 100% | 100% | 100% | 100% |
|  | 2 | 99% | 100% | 100% | 100% | 100% | 100% |
|  | 3 | 97% | 97% | 97% | 98% | 98% | 98% |
|  | 4 | 93% | 93% | 93% | 95% | 95% | 95% |
|  | 5 | 90% | 90% | 92% | 91% | 91% | 92% |
|  | 6 | 91% | 92% | 93% | 90% | 91% | 92% |
|  | 7 | 93% | 94% | 94% | 92% | 93% | 94% |
|  | 8 | 95% | 96% | 96% | 94% | 95% | 95% |
|  | 9 | 96% | 97% | 97% | 96% | 97% | 97% |
|  | 10 | 97% | 98% | 98% | 97% | 98% | 98% |
|  | 11 | 98% | 98% | 98% | 98% | 98% | 98% |
|  | 12 | 99% | 98% | 98% | 99% | 98% | 98% |
|  | 13 | 99% | 99% | 98% | 99% | 99% | 99% |

**Supplemental** **Table S2.** Average weekly healthcare worker (HCW) availability for low and high transmission rates with a 90% vaccination.

| **Transmission**  **Rate** | **Week** | **Policy 1** | **Policy 2** | **Policy 3** | **Policy 4** | **Policy 5** | **Policy 6** |
| --- | --- | --- | --- | --- | --- | --- | --- |
| Low Transmission  Rate | 1 | 100% | 100% | 100% | 100% | 100% | 100% |
|  | 2 | 100% | 100% | 100% | 100% | 100% | 100% |
|  | 3 | 100% | 100% | 100% | 100% | 100% | 100% |
|  | 4 | 100% | 100% | 100% | 100% | 100% | 100% |
|  | 5 | 99% | 99% | 100% | 99% | 99% | 100% |
|  | 6 | 99% | 99% | 99% | 99% | 99% | 99% |
|  | 7 | 99% | 99% | 99% | 99% | 99% | 99% |
|  | 8 | 99% | 99% | 99% | 99% | 99% | 99% |
|  | 9 | 99% | 99% | 99% | 99% | 99% | 99% |
|  | 10 | 99% | 99% | 99% | 99% | 99% | 99% |
|  | 11 | 99% | 99% | 99% | 99% | 99% | 99% |
|  | 12 | 99% | 99% | 99% | 99% | 99% | 99% |
|  | 13 | 99% | 99% | 99% | 99% | 99% | 99% |
| High Transmission  Rate | 1 | 100% | 100% | 100% | 100% | 100% | 100% |
|  | 2 | 100% | 100% | 100% | 100% | 100% | 100% |
|  | 3 | 99% | 99% | 99% | 99% | 99% | 99% |
|  | 4 | 97% | 98% | 98% | 98% | 98% | 99% |
|  | 5 | 97% | 97% | 98% | 97% | 97% | 98% |
|  | 6 | 97% | 97% | 98% | 97% | 97% | 98% |
|  | 7 | 98% | 98% | 98% | 97% | 97% | 98% |
|  | 8 | 98% | 98% | 98% | 98% | 98% | 98% |
|  | 9 | 99% | 99% | 99% | 99% | 98% | 99% |
|  | 10 | 99% | 99% | 99% | 99% | 99% | 99% |
|  | 11 | 99% | 99% | 99% | 99% | 99% | 99% |
|  | 12 | 99% | 99% | 99% | 99% | 99% | 99% |
|  | 13 | 99% | 99% | 99% | 99% | 99% | 99% |
